# The expanding role of mass spectrometry in the field of vaccine development

**DOI:** 10.1002/mas.21571

**Published:** 2018-05-31

**Authors:** Vaneet Kumar Sharma, Ity Sharma, James Glick

**Affiliations:** ^1^ International AIDS Vaccine Initiative (IAVI) New York New York; ^2^ Independent CMC Consultant Paramus New Jersey; ^3^ Novartis Institutes for BioMedical Research East Hanover New Jersey

**Keywords:** envelope protein, glycan, glycopeptide, glycoprotein, IDMS, MALDI‐TOF MS, IMS, RPLC‐MS, vaccines

## Abstract

Biological mass spectrometry has evolved as a core analytical technology in the last decade mainly because of its unparalleled ability to perform qualitative as well as quantitative profiling of enormously complex biological samples with high mass accuracy, sensitivity, selectivity and specificity. Mass spectrometry‐based techniques are also routinely used to assess glycosylation and other post‐translational modifications, disulfide bond linkage, and scrambling as well as for the detection of host cell protein contaminants in the field of biopharmaceuticals. The role of mass spectrometry in vaccine development has been very limited but is now expanding as the landscape of global vaccine development is shifting towards the development of recombinant vaccines. In this review, the role of mass spectrometry in vaccine development is presented, some of the ongoing efforts to develop vaccines for diseases with global unmet medical need are discussed and the regulatory challenges of implementing mass spectrometry techniques in a quality control laboratory setting are highlighted.

## 1 I**NTRODUCTION**

Vaccination is considered one of the most cost effective medical interventions that have contributed significantly to improving public health during the last century. Various types of vaccines have been developed to eradicate infectious diseases that once killed millions of people: the live‐attenuated vaccines (weakened or altered), inactivated vaccines (toxins generated by the bacteria, and not the bacteria themselves) and subunit and conjugate vaccines (segments of the pathogen).^1^, ^2^ Currently, more than eighty vaccines have been licensed for use in the United States (more than 15 viral vaccines are also FDA approved) and several vaccine candidates are in the development pipeline^3^, ^4^ (https://www.fda.gov/BiologicsBloodVaccines/Vaccines/ApprovedProducts/ucm093833.htm). Efforts are ongoing to not only improve existing vaccines but to develop novel vaccines targeting infectious diseases with a high unmet medical need. The global public health community's goal is to develop preventative and therapeutic vaccines for major diseases targets such as human immunodeficiency virus (HIV), Ebola, Zika, Chikungunya, Middle East respiratory syndrome coronavirus (MERS‐CoV), severe acute respiratory syndrome (SARS), West Nile, Lyme disease, Lassa fever, Yellow fever, Nipah virus, respiratory syncytial virus (RSV), influenza, and Dengue fever.^5^, ^6^, ^7^, ^8^ Unfortunately, despite the increased understanding of immunology and greater insight into the viruses responsible for these infections, limited success has been achieved towards development of vaccines for these epidemics.

As the target populations for the vaccines are healthy individuals, pregnant women, or infants, vaccine safety is of paramount importance. Appropriately, the vaccine landscape is changing from traditional vaccine approaches to cost‐effective, highly scalable, and safe recombinant vaccines.^9^, ^10^ Using recombinant DNA technology, antigens are expressed in yeast, *Escherichia coli*, baculovirus expression vector system (BEVS), or mammalian cell lines.^11^, ^12^, ^13^ Recombinant antigens are engineered to mimic the first step of virus attachment to the cell surface which is mediated by specific glycoproteins. The expressed recombinant antigens undergo multiple purification cycles to produce highly purified vaccines.^14^, ^15^

**Figure 1:**
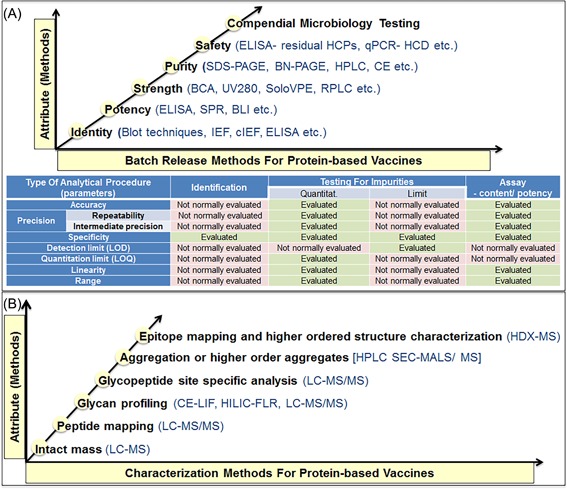
Analytical methods for in‐process and batch release testing of the proteins based vaccines clinical trial material. A, Quality controlled analytical testing for the batch release and stability assessment of the proteins based vaccines and the list of typical parameters which should be considered during the assay validation (or qualification) of analytical method, as per ICH Q2 (R1) guidelines. B, Mass spectrometry based physiochemical characterization testing of the proteins based vaccines

In order for recombinant vaccines to be acceptable to regulatory authorities, in‐depth analytical characterization needs to be performed on the clinical trial material (CTM) to ensure the vaccine is safe and effective. Quality controlled (QC) analytical testing is routinely performed on the clinical trial material during batch release to certify that the content (testing performed by either reversed‐phase high performance liquid chromatography (RP‐HPLC), UV/Vis spectrophotometer [measuring absorbance at 280 nm] or SoloVPE [newest evolution of UV‐Vis spectroscopy]), purity (testing performed by either size exclusion chromatography [SEC], sodium dodecyl sulfate polyacrylamide gel electrophoresis [SDS‐PAGE], or capillary electrophoresis sodium dodecyl sulfate [CE‐SDS] and capillary isoelectric focusing [cIEF]) and residual impurities (testing performed by enzyme‐linked immunosorbent assay [ELISA] for host cell proteins, quantitative polymerase chain reaction [qPCR] for host cell DNA etc.) are within the product specifications and to ensure potency (testing performed by ELISA or ligand free binding assays such as biolayer interferometry [BLI], surface plasmon resonance [SPR]) and safety (microbial enumeration tests, endotoxin testing etc.) of the vaccine candidate (as per ICH harmonised tripartite guideline Q6B, http://www.ich.org). Another regulatory prerequisite is to perform extensive physiochemical characterization of these recombinant antigens. Mass spectrometry is the method of choice to perform the comprehensive physiochemical characterization of glycoproteins. Mass spectrometry‐based characterization is critical not only from the standpoint of understanding the product's structure but also to help establish clinical trial material release specifications and to ensure quality (Figure 1). Thus, innovative vaccine structural design followed by comprehensive analytical characterization is required to successfully develop novel vaccines against epidemic infectious diseases impacting large segments of the global population.

In this review, we focus on the expanding role of mass spectrometry in vaccine development, irrespective of the route of production. We also highlight the regulatory challenges and limitations of mass spectrometry‐based techniques which constrain its further implementation as a quality control batch release assay in cGMP manufacturing.

## 2 ROLE OF MASS SPECTROMETRY IN THE VACCINE DEVELOPMENT

The role of mass spectrometry (MS) has been very limited in the discovery and development of traditional vaccines containing live, attenuated, or inactivated pathogens. However, as recombinant antigens or virus‐like particles (VLPs) are being explored for vaccine development, new opportunities for the use of MS have emerged. Irrespective of the cells used for preparation of the vaccine, the expressed recombinant antigens mimic viral glycoprotein(s) and thus are inherently large molecule weight, structurally complex, and heterogeneous glycosylated proteins. The ease of coupling liquid chromatography (LC) with MS offers exciting opportunities to leverage this analytical platform to obtain detailed structural information about not only its primary structure (the amino acid sequence) but also the molecular weight, disulfide bond linkages, chemical modifications, and post‐translational modifications (PTMs). One of the most abundant and complex protein PTMs is glycosylation which consists of an oligosaccharide linked to an amino acid in the protein. In the case of N‐linked glycans, an asparagine residues at the sequence motif of asparagine‐X‐serine/threonine (where X is any amino acid except proline) is coupled via a nitrogen atom to the oligosaccharide. Alternatively, an O‐linked glycan can be formed by linking the oligosaccharide to a serine or threonine residue. Glycosylation play a fundamental role in antigen conformation, folding, stability, solubility, and importantly, immune response.^16^, ^17^ Plants, yeasts, and non‐human cell lines generate glycans that are not compatible and bioactive within human hosts.^18^ Thus, about 70% of all recombinant glycoproteins are produced in mammalian‐based expression systems such as Chinese Hamster Ovary (CHO) cells.^19^

**Figure 2:**
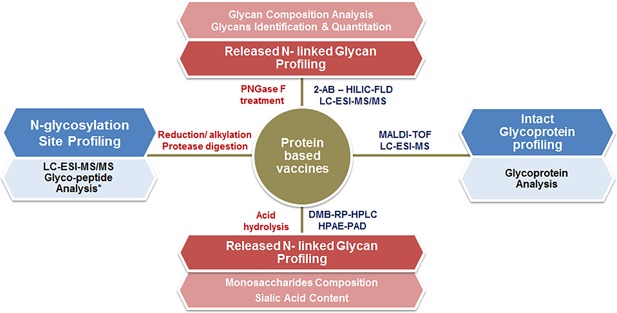
Liquid chromatography and mass spectrometry based major routes for comprehensive analysis of the N‐linked glycans and glycoprotein

Analytical characterization of a glycoprotein is challenging as there is inherent unpredictability associated with the glycans; they are either macroheterogeneous (potential glycan site in the protein not glycosylated) or microheterogeneous (different glycan structures found on the same site in the expressed protein). Glycosylation analysis is performed to understand the nature of structural heterogeneity of glycans, quantify them, and more importantly to determine where glycosylation occurs (site specific analysis). Mass spectrometry based techniques are valuable tool for detecting and investigating glycosylation (Figure 2). Although MS‐based glycosylation analysis is not without limitations, the advances in MS instrumentation and glycan analysis software have led to increased resolution, automated identification, quantitative determination, and accurate structural characterization.^16^, ^20^, ^21^

Another critical quality attribute (CQA) of glycoprotein is intramolecular disulfide bonds (S‐S linkages); they ensure correct folding, functional activity and stability. Incorrect formation of disulfide bonds can cause protein misfolding which tends to promote aggregation which could result in an unwarranted immune response. Confirmation of correct disulfide bond formation in the recombinant protein is ensured by mapping the scrambling of disulfide bonds using LC‐MS/MS‐based methods.^22^ Additionally, LC‐MS/MS‐based methods are being increasingly used for the identification and quantification of host cell proteins (HCPs) for the antigen produced in the CHO cells.^23^

As illustrated in the following section and in Table 1, mass spectrometry‐based techniques have been used to perform the structural characterization, glycosylation profiling and antigen quantitation during the development of the HIV, influenza, Dengue, Ebola, Meningococcal, and other vaccines. The review also highlights that mass spectrometry‐based methods such as glycan analysis has been used to analyze a specific envelope glycoproteins (Env) and has broad applicability to any other glycoprotein‐based vaccines.

**Table 1:** An overview of the role of MS technologies in the vaccine development

Antigen	Study	Technique and instrumentation	Reference
HIV gp120 Recombinant envelope glycoprotein (rgp120) expressed in CHO cells.	First study analyzing the site‐specific N‐glycosylation of gp120. This report describes the structural characterization of the expressed gp120.	Reversed phase HPLC of the tryptic digest. Peptides collected from RP‐HPLC were further identified by amino acid analysis (AAA) or N‐terminal sequencing analysis.	Leonard et al^38^
HIV‐1 gp120 Expressed in CHO Cells.	Mass spectrometric characterization of the glycosylation pattern. Of HIV‐1 gp120.	MALDI and nanoESI‐LC‐MS/MS using a hybrid quadrupole‐time‐of‐flight tandem mass spectrometer (Q‐TOF) was used to assign glycosylation sites.	Zhu et al^111^
HIV gp140 JR‐FL and CON‐S Env expressed in CHO and HEK293 cells.	A glycopeptide‐based mass mapping approach was used to characterize the glycosylation of two Env protein vaccine candidates in a glycosylation site‐specific fashion.	Trypsin digested HIV envelope glycoprotein was either subjected to off‐line RP‐HPLC fractionation for MALDI or RP‐HPLC/ESI‐FTICR MS analyses.	Go et al^41^
HIV gp120/gp41 C.CON and C.97ZA012 Env expressed in HEK 293T cells.	Mass spectrometry‐based glycosylation profiling of two rVV expressed clade C HIV‐1 Envs was performed.	Trypsin digested HIV envelope glycoprotein was either subjected to off‐line RP‐HPLC fractionation for MALDI or RP‐HPLC/ESI‐FTICR MS analyses.	Go et al^41^
HIV gp120 Expressed in HEK293 cells.	Ion mobility ESI‐MS was used to perform the analysis of N‐glycans from engineered glycoforms of intact, folded HIV gp120.	IMS‐MS was performed on a Synapt G2 mass spectrometers. ESI‐MS/MS was performed with a Waters Q‐TOF in negative ion mode.	Harvey et al^57^
HIV gp140 B.700010040.C9 and C.1086 Env expressed in HEK293T cells.	Glycopeptide‐based mass mapping approach was used to perform site specific glycosylation analysis of the transmitted/founder Envs.	LC/ESI‐MS was performed using a Thermo Scientific LC‐ESI‐LTQ‐FTICR MS directly coupled to the Dionex UltiMate capillary LC system equipped with a FAMOS well plate autosampler.	Go et al^42^
HIV gp140 JRFL gp140 ΔCF Env expressed in HEK 293 cells.	LC‐MS/MS based comprehensive analysis of a partially deglycosylated HIV vaccine candidate Env using PNGase F, Endo H and Endo F3 glycosidases.	LC/ESI‐MS was performed using a Thermo Scientific LC‐ESI‐LTQ‐FTICR MS directly coupled to the Dionex UltiMate capillary LC system equipped with a FAMOS well plate autosampler.	Go et al^33^
HIV gp140 CN54gp140 Env expressed in CHO cells.	Comprehensive mass spectrometric analysis of the site‐specific glycosylation of gp120.	LC‐ESI‐MS was performed using an ion trap as well as a Q‐TOF instrument and standard software for glycopeptide identification.	Pabst et al^112^
HIV gp120 1086.C Env expressed in CHO and HEK293 cell lines.	Evaluated and compared the host‐cell specific glycosylation pattern of recombinant HIV‐1 gp120 expressed in CHO and 293T cell lines.	An integrated glycopeptide‐based LC‐MS/MS mapping workflow that includes a partial deglycosylation step, fragmentation techniques (ETD and CID) was carried out using a hybrid LC‐ESI‐LTQ‐FTICR MS.	Go et al^45^
HIV gp120 Expressed in HEK293 and ACH‐2 cell lines.	LC‐MS/MS based site‐specific glycosylation profiling of recombinant gp120 was performed using a novel spectral‐aligning strategy.	MS analysis was performed using a Thermo Q Exactive mass spectrometer *via* higher energy collisional dissociation and spectral‐aligning strategy.	Yang et al^21^
HIV gp120 C97ZA012 gp140 trimeric Env expressed in HEK293T cell lines (transiently & stably expressed).	Mass spectrometry based glycosylation and disulfide bond analysis of transiently and stably expressed clade C HIV‐1 gp140 trimers in 293T cells.	High‐ and low‐resolution LC–MS experiments were performed using two different platforms; LC‐ESI‐LTQ‐FTICR MS coupled to a nanoAcquity UPLC system and the second was an LTQ Velos mass spectrometer with ETD (ThermoScientific) coupled to Acquity UPLC system (Waters).	Go et al^46^
HIV trimers Soluble native SOSIP.664 trimers (92UG037.8 and CZA97.012 clade A and clade C env genes) expressed in HEK293F cells.	Comprehensive mass spectrometric analysis of the native SOSIP.664 trimers.	LC/MS based glycosylation analysis and HDX‐MS was performed using Waters Synapt G2Si MS. LC‐ESI‐MSMS based disulfide mapping was performed using a LTQ Orbitrap Velos Pro (Thermo Scientific).	Ringe et al^47^
HIV Env trimers HIV‐1 JR‐FL Env Δ712 and Env Δ808 expressed in CHO cells	MS‐based glycosylation analysis was used to evaluate whether Env glycosylation is dependent on the Env form, ie, membrane anchored or soluble.	High‐resolution LC‐MS/MS was performed using an Orbitrap Velos Pro hybrid mass spectrometer (Thermo Scientific) coupled to an Acquity UPLC system (Waters).	Go et al^113^
HIV gp120 JRCSFBG505 Env expressed in HEK293T cells.	Env glycosylation analysis by ion mobility mass spectrometry.	Ion mobility experiments were carried out with a Waters Synapt G2 traveling wave ion mobility mass spectrometer fitted with an ESI ion source.	Harvey et al^59^
HIV gp120 Gp120SU Env expressed in BaL/SUPT1‐R5 cell line [CLN204].	A study of the site‐specific N‐glycosylation of virion‐derived gp120 by mass spectrometry showing the dominance of oligomannose‐type glycans on almost all N‐glycosylation sites.	MALDI‐MS/MS was performed using Applied Biosystems 4800 MALDI‐TOF/TOF mass spectrometer operated in reflector positive‐ion mode. Nano‐LC‐ES‐MS/MS analysis was performed using HPLC system connected to a Q‐TOF (API Q‐STAR^®^ Pulsar I, Applied Biosystems/MDS Sciex).	Panico et al^114^
HIV trimers BG505 SOSIP.664 trimers expressed in stable HEK293T cell line.	A parallel mass‐spectrometric‐based approach exploiting two different ionization modes was used to perform quantitative, site‐specific N‐glycosylation analysis to understand the glycan shield of the BG505 SOSIP.664 trimer.	IM‐ESI MS with a Waters Synapt G2Si mass spectrometer and RP‐HPLC separated glycopeptide pools were analyzed using an Autoflex Speed MALDI‐TOF/TOF (Bruker). Enriched glycopeptides were also analyzed on a Q‐Exactive Orbitrap mass spectrometer coupled to a Dionex Ultimate 3000 nanoLC system.	Behrens et al^51^
HIV gp120 1086.C and TV1.C Env expressed in stable CHO cell lines.	Mass spectrometry based characterization of a bivalent HIV‐1 Subtype C gp120 protein boost for proof‐of‐concept HIV vaccine efficacy trials in Southern Africa.	High‐ and low‐resolution LC–MS experiments were performed using two different platforms; LC‐ESI‐LTQ‐FTICR MS coupled to a nanoAcquity UPLC system and the second was an LTQ Velos mass spectrometer with ETD (ThermoScientific) coupled to Acquity UPLC system (Waters).	Zambonelli et al^48^
HIV gp120 1086.C and TV1.C Env expressed in stable CHO cell line.	Comprehensive characterization of reference standard lots of HIV‐1 subtype C Gp120 proteins for clinical trials in southern African regions (HVTN 100 Phase I/II clinical trial material and HVTN 702 Phase IIb/III clinical trial material).	Intact molecular weight was measured using Bruker UltrafleXtreme MALDI‐TOF/TOF instrument. Mol. Weight of de‐N‐glycosylated was determined by LC‐MS using a Waters Xevo G2‐S QTOF. N‐Linked glycosylation site mapping was carried out by analyzing the reduced and alkylated tryptic peptides digested by Endo H, Endo F3, or PNGase F by LC‐MS/MS using a Thermo LTQ Orbitrap MS. LC‐MS/MS analysis for the disulfide mapping was carried out using LTQ Orbitrap with both CID and ETD.	Wang et al^63^
HIV trimers Eleven different trimeric Env expressed in CHO and HEK293 cells.	Mass spectrometry based approach was used to map the complete glycosylation profile at every site in eleven HIV‐1 Env trimers.	High‐resolution LC‐MS/MS was performed using an Orbitrap Velos Pro hybrid mass spectrometer (Thermo Scientific) equipped with ETD and coupled to an Acquity UPLC system (Waters).	Go et al^49^
HIV gp120 Monomers of the BG505.SOSIP.	Global N‐Glycan site occupancy of HIV‐1 gp120 by metabolic engineering and high‐resolution intact mass spectrometry was performed.	Released N‐glycan analysis was carried out using Synapt G2Si ion mobility mass spectrometer. Native high‐resolution mass spectrometry was performed on Q Exactive hybrid Quadrupole‐ Orbitrap mass spectrometer.	Struwe et al^60^
HIV trimers JR‐FL SOSIP.664 (subtype B), B41 SOSIP.664 (subtype B), CRF02_AG_250 SOSIP.664 (subtype AG), 327c SOSIP.664 (subtype C), and BG505 SOSIP.664 trimer (subtype A). All of these Env were expressed in HEK293F cells.	Global site‐specific N‐glycosylation analysis of HIV‐1 Env was performed. This study presents a specific endoglycosidases strategy for MS analysis.	Mass spectrometric analysis was performed on Fusion Orbitrap tribrid mass spectrometer (Thermo Fisher Scientific).	Cao et al^62^
HIV trimers BG505 SOSIP.664 expressed in stable CHO cell line.	Mass spectrometry based testing and N‐glycosylation characterization performed on the cGMP‐quality BG505 SOSIP.664 trimers.	N‐linked glycosylation analysis was carried by HILIC‐UPLC method and the disulfide bonds were determined by LC‐MS/MS analysis using Q Exactive™ hybrid quadrupole‐Orbitrap™ mass spectrometer.	Dey et al^21^
Influenza A/New Caledonia/20/99 A/Panama/2007/99 A/Wyoming/3/2003	HA quantification and identification of influenza A&B strains propagated in PER.C6^®^ cells.	RP‐HPLC performed using Waters Alliance 2695 system and a POROS^®^ R1/10 (2.1 mm × 100 mm) column (PerSeptive Biosystems Inc.) maintained at 65 °C and UV detection at 214 nm.	Kapteyn et al^115^
Influenza A/New Caledonia/20/99 B/Jiangsu/10/2003	Selective and quantitative detection of influenza virus proteins in commercial vaccines.	2D‐HPLC (on‐line coupling of size exclusion HPLC to reversed‐phase HPLC).	Garcia‐Canas et al^81^
Influenza A/Puerto Rico/8/34 (PR8) A/Vietnam/1203/2004/PR8‐RG2 A/Vietnam/1203/2004	Quantification of influenza virus hemagglutinins in complex mixtures. HA from viral subtypes H1, H3, H5, and B was determined both directly and rapidly.	Isotope dilution tandem mass spectrometry (IDMS) and a multiplexed multiple reaction monitoring (MRM) approach) was performed using a Symmetry300 reverse phase C18 column and Thermo *Quantum* TSQ mass spectrometer with an electrospray interface.	Williams et al^70^
Influenza Trivalent bulk vaccines (TBV) from Northern Hemisphere (NH) season 2006/2008; A/Hiroshima/52/2005 A/New Caledonia/20/99 B/Malaysia/2506/2004 Season 2007/2008; A/Wisconsin/67/2005 A/Solomon Islands/3/2006 B/Malaysia/2506/2004	HA quantification performed for the active or formaldehyde‐inactivated egg‐based and MDCK cell‐based whole virus samples.	RP‐HPLC performed using Waters Alliance 2695 system using a polystyrene POROS^®^ R1/10 (2.1 mm × 100 mm) column (PerSeptive Biosystems Inc.) maintained at 65 °C and UV detection at 214 nm.	Kapteyn et al^116^
Influenza	Optimization of digestion parameters for protein quantification.	Isotope dilution tandem mass spectrometry (IDMS) and a multiplexed multiple reaction monitoring (MRM) approach) was performed using Thermo *Quantum* TSQ mass spectrometer.	Norrgran et al^71^
Influenza	A combination of separation and identification techniques was used to rapidly and reproducibly analyze influenza vaccine constituents.	SEC‐HPLC analysis was performed, peaks were collected and underwent tryptic digest followed by MALDI/MS.	Garcia‐Canas et al^82^
Influenza Monovalent bulks: A/Brisbane/59/2007 H1N1 A/Brisbane/59/2007 H1N1 A/California/07/2009 H1N1 A/Uruguay/716/2007 B/Florida/4/2006	Reversed‐phase HPLC method was developed and optimized for the quantitative determination of HA in influenza vaccine preparations.	RP‐HPLC was performed using a Waters Alliance 2695 coupled to fluorescence detector (λ_ex_ 280 nm and λ_em_335 nm) and a UV–vis photodiode array detector. MICRA^®^ HPLC NPS‐ODSI, 4.6 mm × 33 mm, column was used and chromatographic separations were carried out at 60 °C.	Lorbetskie et al^117^
Influenza Hemagglutinins (HA) in trivalent influenza vaccines (TIV).	Quantification of immunoreactive viral influenza proteins using an immunocapture isotope dilution mass spectrometry (IC‐IDMS) method.	The captured proteins were digested, and evolutionarily conserved tryptic peptides were quantified using IDMS‐MRM approach performed using Thermo *Quantum* TSQ mass spectrometer with an electrospray interface.	Pierce et al^75^
Trivalent influenza vaccine (Protein Sciences Corp., Meriden, CT). A/Brisbane/59/2007 (H1N1), A/Brisbane/16/2007 (H3N2) B/Florida/4/2006	LC‐MS was performed for simultaneous identification of HA proteins and process‐related impurities in a trivalent influenza candidate vaccine, comprised of purified recombinant HA (rHA).	LC‐MS^E^ measurements were performed on a Waters Synapt HDMS system coupled with a Waters ACQUITY UPLC for online RP LC separation. The detected site‐specific glycoforms were further confirmed and quantified by HILIC‐ MRM assays.	Xi et al^118^
Influenza Commercial 2007/2008 vaccine; A/Solomon Islands/3/2006 A/Wisconsin/67/2005 B/Malaysia/2506/2004‐like strains Commercial 2009/2010 vaccine; A/Brisbane/59/2007 A/Uruguay/716/2007 B/Brisbane/60/2008‐like strains. Commercial 2010 vaccine; A/California/7/2009 A/Perth/16/2009 B/Brisbane/60/2008‐like strains.	LC/MS/MS method was performed for the absolute quantification of viral proteins in a complex mixture.	IDMS and MRM approach was performed using a Symmetry300 reverse phase C18 column and Thermo *Quantum* TSQ mass spectrometer with an electrospray interface.	Williams et al^72^
Influenza Commercial 2007/2008 vaccine; A/Solomon Islands/3/2006, A/Wisconsin/67/2005 B/Malaysia/2506/2004‐like strains Commercial 2009/2010 vaccine; A/Brisbane/59/2007 A/Brisbane/10/2007 B/Brisbane/60/2008‐like strains. Commercial 2010 vaccine; A/California/7/2009, A/Victoria/210/2009, and B/Brisbane/60/2008‐like strains.	Simultaneous quantification of the viral antigens HA and NA in influenza vaccines. The central premise of MS^E^ quantification is that the “top 3” most intense peptide ions in an LC–MS chromatogram are approximately equal for all proteins at equimolar concentrations.	LC–MS^E^ was performed using nanoAcquity UPLC BEH130 C18 reverse‐phase analytical column (100 μm × 100 mm). The eluting peptides were analyzed with a Waters Synapt HDMS system operating in MS^E^ mode.	Creskey et al^83^
Influenza A/Brisbane/59/2007 A/Solomon Islands/03/2006 A/New Caledonia/20/1999 A/Vietnam/1203/2004 B/Brisbane/60/2008 monovalent 2009 H1N1 vaccine	Quantification of recombinant HA and NA in influenza virus using a label‐free mass spectrometry (MS) based method that enables simultaneous identification and quantification of HA, NA, and other viral proteins.	Samples were analyzed by LC/MS^E^ using a nanoAcquity UPLC and Synapt G2 mass spectrometer equipped with a NanoLockSpray ion source (Waters).	Getie‐Kebtie et al^78^
Influenza A/Vietnam/1203/2004 (H5N1) expressed in three different insect cell lines. A/Vietnam/1203/2004 (H5N1) virus cultivated in hen eggs A/bar‐headed goose/Qinghai/14/2008 (H5N1) produced in HEK293	A method using nanoLC/MSE and quantitative MALDI‐TOF MS permethylation was developed to monitor the glycosylation of HA's from two different influenza H5N1 strains produced in five different platforms, including hen eggs, three different insect cell lines (High Five™, expresSF + ^®^ and glycoengineered expresSF +), and a human cell line (HEK293).	Mass spectrometry analysis was performed using a BEH C18 column on Waters Synapt G2 HDMS system. MALDI‐TOF analysis of permethylated N‐glycans was performed using a Perseptive Biosystems Voyager DE RF MALDI‐TOF mass spectrometer. Samples were analyzed in positive ion reflectron mode in the 800–5500 *m/z* range.	An et al^119^
Influenza A/Netherlands/219/2003 (H7N7) A/Shanghai/2/2013 (H7N9)	An accurate and precise IDMS method was used to quantify the HA and the NA proteins in a purified virus preparation of A/Netherlands/219/2003 (H7N7) in the same analytical run.	IDMS was performed using Agilent Technologies 1200 HPLC using a Symmetry300 C18 Waters column. The eluent was introduced into a Thermo Scientific Vantage TSQ triple quadrupole tandem mass spectrometer with an electrospray interface.	Santana et al^73^
Influenza A/Hong Kong/1/68 (H3N2: HK68)	HA N‐Glycosylation analysis of engineered H3N2 virus strain was carried out by mass spectrometry based approaches.	Nano‐LC‐MS^E^ was used for glycopeptide composition, sequence and site occupancy analysis, and MALDI‐TOF MS permethylation profiling carried out for characterization of released HA N‐glycans.	An et al^120^
Influenza A/Puerto Rico/8/1934 (H1N1) A/Aichi/2/1968 (H3N2) A/Wilson‐Smith/1933 (H1N1) A/Hong Kong/8/1968 (H3N2) and B/Lee/1940	IEX‐HPLC method for the quantification of viral influenza particles is presented.	IEX‐HPLC was performed using the Alliance HPLC system equipped with a 2695 separations module, 2475 fluorescence detector and Empower™ software. A monolithic column (5.2mm × 5.0 mm) was used to separate the virus.	Transfiguracion, et al^121^
Influenza Trivalent influenza vaccines (2011–2012 and 2014–2015) H1N1 H3N2	Quantification of the antigens HA and NA in influenza vaccines has been reported using an antibody‐free LC–MS based method known as MS^E^ “Hi3”.	MS^E^ Hi3 was performed using a Waters nanoAcquity UPLC with a BEH130 C18 reverse‐phase column (100 µm × 100 mm) and peptide analysis was carried out by a Waters Synapt HDMS system operating in data independent analysis mode.	Smith et al^122^
Influenza A/California/7/2009 (H1N1 A/California/7/2009 (H1N1)	Evaluation of HA content by RP‐HPLC to generate pandemic influenza vaccine.	Waters Alliance 2695 system using a polystyrene POROS R1/10 (2.1 mm × 100 mm) column and Waters 2996 PDA detector. A UV detector at 214 nm was used.	Kang et al^123^
Influenza A/Shanghai/2/2013	IC‐IDMS was performed to evaluate the suitability of the underlying monoclonal and polyclonal antibodies for their capacity to isolate the H7 hemagglutinin in the vaccine for quantification by IDMS.	IDMS was performed using Agilent Technologies 1200 HPLC using a Symmetry300 reverse phase C18 Waters column. The eluent was introduced into a Thermo Scientific Vantage TSQ triple quadrupole tandem mass spectrometer with an electrospray interface.	Pierce et al^76^
Influenza A/California/7/2009	Topological N‐glycosylation and site‐specific N‐glycan sulfation of influenza proteins in the highly expressed H1N1 candidate vaccines.	High‐resolution LTQ‐FT and Orbitrap mass spectrometer was used to identify the N‐glycan structure of intact glycopeptides of the protein digests by low‐energy collision‐induced dissociation followed by multi‐stage tandem mass spectrometry (MS^3^).	She et al^124^
Influenza A/Mallard/Denmark/64650/03 (H5N7).	Glycosylation patterns of four recombinant H5 hemagglutinins derived from H5N7 were characterized using mass spectrometry based methods.	Two semi quantitative analyses were performed; MALDI‐TOF MS permethylation analysis for the released glycans and LC–MS^E^ analysis for glycosylation profiling and site occupancy.	Parsons et al^125^
Influenza	Fast and highly selective determination of hemagglutinin content in quadrivalent influenza vaccine by RP‐HPLC method.	HPLC analysis was performed using a Waters Alliance2695 chromatograph coupled to a 2475 Fluorescence and a 2996 UV–vis photodiode array detector. Chromatographic separation of HA in QIV formulations was achieved by using a MICRA^®^HPLC NPS‐ODSI, 33 mm × 4.6 mm, column at 55 °C by using 0.04% (v/v) aqueous TFA as eluent A and 0.03% (v/v) TFA in 25% ACN and 75% 2‐propanol as eluent B.	Lorbetskie et al^85^
Influenza A/Victoria/361/2011 (H3N2)	N‐linked glycosylation analysis was performed for the recombinant influenza virus haemagglutinin (HA) from H3N2 strain produced in HEK 293 F cells.	Glycosylation site occupancy of H3N2 strain was performed using a Fusion Orbitrap tribrid mass spectrometer (Thermo Fisher Scientific). A reversed phase BEH C18 column was used to perform chromatographic separation.	Cao et al^62^
Meningococcal vaccine CRM_197_ and meningococcal serogroups A (MenA), C (MenC), W135 (MenW) and Y (MenY) glycoconjugates	Mass spectrometry based characterization of glycoconjugate molecules designed to prepare a vaccine against *Neisseria meningitides* serogroups A, C, W135 and Y was performed.	SEC–MS analysis was performed using a Superdex Peptide PC 3.2/30, and LC‐MS/MS analysis was performed using Vydac C4 column on Q‐TOF (Micromass).	Bardotti et al^97^
Meningococcal vaccine	Semi quantitative LC‐MS/MS analysis carried out to define conjugation of glycans to the lysines of Cross‐Reactive‐Material‐197 (CRM_197_). Conjugate vaccines use CRM_197_ as carrier protein.	NanoLC‐ESI‐MS analysis was carried out on an Ultimate 3000 RSLC‐nano system (Dionex/Thermo Scientific) coupled to an amaZon speed ETD ion trap (Bruker). LC‐MALDI‐TOF‐MS experiments were performed on an ultrafleXtreme MALDI‐TOF/TOF (Bruker).	Crotti et al^98^
Meningococcal group B Vaccine (Bexsero(^®^)	BEXSERO is a FDA approved *Neisseria meningitidis* serogroup B (MenB) vaccine licensed for active immunization to prevent invasive disease caused by MenB. Quantitative proteomics for the vaccine was performed.	Quantification of outer membrane vesicle proteins of the Bexsero^®^ vaccine was performed using 1.7 µm BEH130 C18 analytical column (75 µm × 250 mm, Waters) and nanoAcquity UPLC coupled to a Synapt G2 mass spectrometer. Hi3 methodology was used to perform quantitative proteomics.	Tani et al^100^
Meningococcal group B vaccine	Label‐free quantitative LC‐MS/MS analysis of protein antigens in a meningococcal group B outer membrane vesicle vaccine was carried out.	MRM‐based LC‐MS/MS was carried out using a Waters BEH C18 column and 4000 Q‐Trap mass spectrometer.	Dick et al^96^
Meningococcal group B vaccine Trumenba (bivalent rLP2086)	Mass spectrometry based characterization of the bivalent rLP2086 vaccine (Trumenba^®^), a *Neisseria meningitidis* serogroup B (MenB) vaccine was carried out.	GC/MS was used to analyze the composition of fatty acids released from rLP2086‐A05 and rLP2086‐B01. LC‐MS/MS analysis was carried out a Waters BEH C4 column at 60 °C using UHPLC/UV interfaced to an ultrahigh‐resolution Bruker Daltonics maXis ESI‐QTOF to characterize the primary sequence and PTMs.	Luo et al^101^
Meningococcal vaccine	Glycoconjugates vaccines consisting of CRM_197_ and synthetic oligosaccharide epitopes was characterized using mass spectrometry techniques.	The primary structure was assessed by combining intact protein MALDI‐TOF‐MS, LC‐MALDI‐TOF‐MS middle‐down and LC‐ESI‐MS bottom‐up approaches.	Möginger et al^126^
Multicomponent meningococcal serogroup B vaccine (4CMenB; Bexsero^®^)	The active components of the Bexsero vaccine; Neisseria heparin binding antigen, factor H binding protein, Neisseria adhesion A, and outer membrane vesicles were separated and analyzed.	A fast, selective and sensitive UHPLC method for the determination of the Bexsero antigens in the vaccine supernatant is presented in this study.	Nompari et al^127^
Gonorrhea Vaccine	A comprehensive proteomic platform − isobaric tagging for absolute quantification coupled with 2D‐LC‐MS was used to characterize potential gonococcal vaccine antigens.	2D LC‐MS/MS was performed after the cell envelope‐associated proteins were precipitated, trypsinzed and labeled with iTRAQ reagents. Desalted SCX fractions were analyzed by LC/ESI MS/MS with a ThermoScientific nano HPLC coupled to a hybrid Orbitrap Elite ETD mass spectrometer.	Zielke et al^128^
Dengue vaccine Sanofi Pasteur tetravalent (CYD). Four chimeric viruses produced in mammalian Vero cells	Mass spectrometry based site‐specific characterization of envelope protein N‐glycosylation was performed. First report assessing the specific N‐glycosylation pattern of the E‐protein using mass spectrometry.	N‐glycosylation profiling of the E‐protein was performed using MALDI‐TOF (Ultraflextreme MALDI‐TOF‐MS in reflectron positive mode for neutral glycans and in negative mode for acidic glycans). The characterization of sitespecific N‐glycosylation was assessed by nanoLC–ESI‐MS/MS using a Bruker Maxis Q‐TOF mass spectrometer or an ion trap HCT.	Dubayle et al^87^
Ebolavirus glycoprotein Recombinant Ebola glycoprotein expressed in HEK 293 cells	Comprehensive characterization of glycosylation was done for EBOV Yambuku GP1.	Identification of N‐glycans from Ebola virus glycoproteins by matrix‐assisted laser desorption/ionisation time‐of‐flight and negative ion electrospray tandem mass spectrometry.	Ritchie et al^89^
Ebolavirus‐like particles (EBOV VLPs, eVLPs) Recombinant Ebola glycoprotein expressed in HEK 293 cells	GP_1_ concentration is a critical quality attribute of EBOV vaccines and LC‐HRMS was used to perform quantitation of GP_1_ in eVLP vaccine preparations.	AQUA Ultimate (heavy and light) peptides were used to perform LC‐MS/MS analysis using ultimate 3000 HPLC and Orbitrap Elite mass spectrometer with a HESI‐2 ion source (Thermo Fisher Scientific).	Cazares et al^91^
Ebolavirus glycoprotein Recombinant Ebola glycoprotein expressed in HEK 293 cells	The study describes and compares the N‐linked and O‐linked glycosylation patterns for GP1,2 of five pathogenic ebolaviruses (BDBV, SUDV, TAFV, and two EBOV variants).	N‐ and O‐glycan profiling of permethylated glycans by MALDI TOF was performed. Positive ion reflectron MALDI‐TOF mass spectra were acquired using an Autoflex III mass spectrometer (Bruker Daltonics).	Collar et al^90^
Ebolavirus glycoprotein Recombinant Ebola glycoprotein expressed in in insect (Sf9) and HEK 293 cells	The study describe and compare the N‐linked and O‐linked glycosylation patterns for GP1,2 expressed in HEK293 and insect (Sf9) cells.	Glycan compositions and N‐glycan site occupancy was determined after MALDI‐TOF‐MS analysis using 2,5‐dihydroxybenzoic acid as matrix. Positive ion reflectron MALDI‐TOF/TOF mass spectra were acquired using an Autoflex III mass spectrometer (Bruker Daltonics).	Clarke et al^129^
Chikungunya (CHIKV) virus‐like particle (VLP) vaccine.Recombinantly expressed in HEK293 and SfBasic cells	A sensitive RP‐ HPLC method that separates capsid, E1, and E2 proteins in CHIKV VLP vaccine with good resolution was developed to characterize and quantitate CHIKV VLP components.	HPLC separation was performed using XBridge BEH300 C4 column held at 60 °C. MALDI‐TOF was performed to characterize the separated proteins using Bruker Autoflex III operated in reflector mode. The PTMs on the viral glycoproteins E1 and E2 were further identified by LC‐MS using a Synapt G2 mass spectrometer.	Shytuhina et al^92^
Chikungunya (CHIKV) virus‐like particle (VLP) vaccine.Recombinantly expressed in HEK293 and **Sf**Basic cells	The glycosylation patterns of CHIKV virus‐like particles (VLPs), containing both E1 and E2 proteins, derived from mammalian and insect cells were characterized.	HILIC with fluorescence and mass spectrometry (MS) based characterization of N‐glycosylation profiles from mammalian and insect cell derived chikungunya VLP was performed.	Lancaster et al^93^

### 2.1 Human immunodeficiency virus (HIV)

The quest for a safe and effective vaccine to protect against human immunodeficiency virus type 1 (HIV‐1) infection is ongoing.^24^, ^25^, ^26^, ^27^, ^28^, ^29^, ^30^ Even after thirty five years of the HIV epidemic, there is no vaccine approaching licensure. An estimated 36.7 million people globally were living with HIV in 2016, with approximately 1.8 million new infections diagnosed in 2016. Despite major advances in antiretroviral therapy (ART) against HIV‐1, an inspired global commitment will be required to end the epidemic by 2030 (http://www.unaids.org/en/resources/fact-sheet). Unfortunately, developing an effective vaccine has been very challenging for reasons related to the nature of the HIV‐1 virion.^31^ Of several vaccine concepts tested in efficacy trials, only one, the RV144 pox virus prime, protein boost (ALVAC/AIDSVAX B/E) vaccine, showed a low level of vaccine protection with an estimated 31% vaccine efficacy.^32^

One of the hypotheses being explored for protective immunity against HIV is to induce broadly neutralizing antibodies (bNAbs) that target the highly glycosylated HIV‐1 virion‐associated Envelop.^33^ A number of approaches are being pursued in the HIV‐1 vaccine development field to establish protective immunity, including monomeric gp120 subunits and oligomeric gp140 and trimeric envelope glycoprotein to elicit bNAbs.^34^, ^35^, ^36^ Each of these Envelop (Env) immunogens have multiple exposed epitopes and are heterogeneously glycosylated molecules, with more than 50% of their mass consisting of glycans (∼28 N‐linked glycans). It is critically important to monitor this HIV Env glycan shield during vaccine development.^37^ A number of methods are available to perform the glycan characterization, but hydrophilic‐interaction liquid chromatography (HILIC) in conjunction with both positive and negative ion mode MS is the most routinely used method for the glycan profiling. LC‐MS/MS using multistage fragmentation techniques such as collision induced dissociation (CID) and electron transfer dissociation (ETD) for the analysis of proteolytically digested glycoproteins is the most commonly used method to determine the site of glycosylation.

The first study to analyze the N‐linked glycosylation of recombinant gp120 which was expressed in CHO cells was performed by fractionating the tryptic digest by reversed phase HPLC with the peptide fraction amino acid sequences subsequently identified by amino acid analysis (AAA) or N‐terminal sequencing.^38^ Extensive analysis of the Env immunogens using MS‐based methods was carried out by Desaire and collaborators. In the last decade, that group demonstrated broadly applicable methods to analyze glycosylation and disulfide mapping of the HIV Env protein. The developed methods have been successfully used to analyze multiple immunogens; gp120 monomers, gp140 oligomers, gp140 trimers, BG505.SOSIP native trimers. MS experiments conducted and results reported by Desarie and collaborators have significantly contributed to advance the development of a HIV vaccine.^39^, ^40^, ^41^, ^42^, ^43^, ^44^, ^45^, ^46^, ^47^, ^48^ Recently, Go et al used a MS‐based approach to map the complete glycosylation profile at every site on eleven trimeric Envs to accomplish two goals. (i) To determine which glycosylation sites contain conserved glycan profiles across different trimeric Envs. (ii) To identify the variables that impact Env's glycosylation profile at sites with divergent glycosylation. The study concluded that the processing of many glycosylation sites on recombinant Envs are not affected by the expression system.^49^

**Figure 3:**
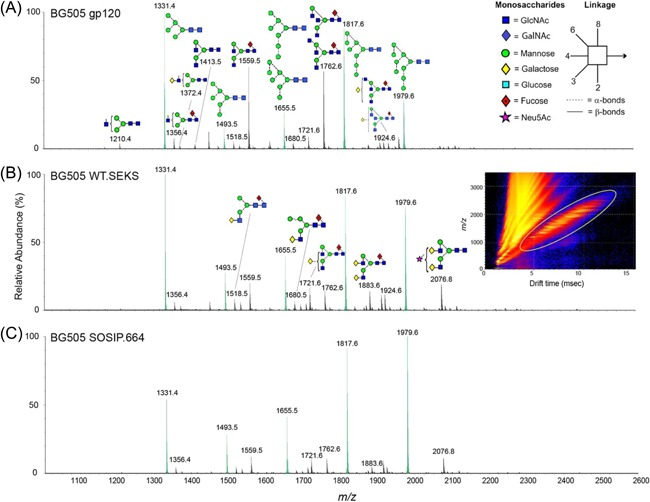
Ion mobility mass spectrometry analysis of BG505 Env glycoproteins. Mobility‐extracted singly charged negative‐ion electrospray spectra are shown for N‐linked glycans found on the following (A) BG505 gp120 monomers, (B) WT.SEKS pseudotrimers (The WT.SEKS construct differs from SOSIP.664 in that it lacks the stabilizing SOSIP mutations and contains an inactivated furin cleavage site (RRRRRR mutated to SEKS); the resulting uncleaved pseudotrimers have a nonnative conformation), and (C) BG505 SOSIP.664 trimers. The inset in panel B shows an example of a DriftScope image derived from gp120 monomers, with singly charged ions encircled with a yellow oval. The peaks of the oligomannose series Man_5‐9_GlcNAc_2_ are highlighted in green. Reprinted from Behrens et al, Journal of Virology, 2017, *91*(2), pp. e01894‐16, Copyright (2018), with permission from Elsevier

Crispin and collaborators also extensively investigated the native glycosylation profile of Envs, in particularly, trimeric immunogen BG505 SOSIP.664.^50^, ^51^, ^52^, ^53^ Their studies led to an increased understanding of the glycan shield of the Env and it is now largely accepted that to induce bNAbs, the HIV vaccine candidate should have a glycan profile similar to the one present on the native Env trimers.^54^, ^55^, ^56^ In a thorough study on trimeric Env glycosylation, Behrens et al^51^ employed a parallel MS‐based approach exploiting MALDI‐TOF MS and LC‐MS/MS to analyze site‐specific glycosylation of a soluble, recombinant trimer BG505 SOSIP.664, resulting in the identification of 20 out of 28 N‐linked glycans.^51^ In a separate set of studies, Harvey et al utilized ion mobility mass spectrometry‐based techniques to analyze Env glycosylation (Figure 3). The ability of ion mobility to separate the isomeric N‐linked glycan was utilized to profile complex type, high‐mannose isomers in the trimeric envelop.^57^, ^58^, ^59^, ^60^, ^61^

**Figure 4:**
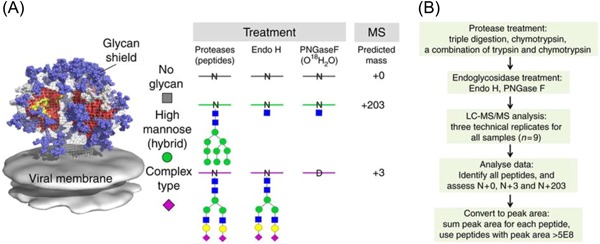
Global site‐specific N‐glycosylation analysis of HIV envelope glycoprotein is presented in this study. A, Introduction of novel masses for peptides with glycosites that contain high‐mannose (hybrid) glycans, complex‐type glycans, or are not glycosylated, by using Endo H treatment followed by PNGase F deglycosylation. B, The workflow of the method present in this study. Reprinted from Cao et al, Nature Communications, 2017, 8, pp.14954, an open access article distributed under the terms of the Creative Commons CC BY license, Copyright (2018), with permission from Springer Nature.

More recently, Paulson and collaborators reported MS‐based site‐specific N‐glycosylation analysis of six different strains of HIV Env glycoprotein. They presented a general MS proteomics strategy that uses specific endoglycosidases to introduce mass signatures that distinguished glycopeptides that were unoccupied or occupied by high‐mannose/hybrid or complex‐type glycans. The method workflow included protease treatment, sequential glycosidase digestion (first treated with Endo H and then digested with PNGase F in O^18^‐water) followed by LC‐MS/MS analysis (Figure 4). The authors determined that almost all of the glycan sites were occupied for the soluble Env trimers. The reported method has the potential to serve as a robust tool to facilitate the rational design and development of vaccine immunogens.^62^

Two HIV‐1 subtype C gp120 Envs (1086.C and TV1.C) are being currently evaluated in a pivotal Phase IIb/III efficacy trial in South Africa (HVTN702) to assess if HIV infection among adults can be prevented. This trial was designed to confirm and extend the partial protection seen against HIV‐1 infection in the RV144 Thai trial (https://www.niaid.nih.gov/news-events/first-new-hiv-vaccine-efficacy-study-seven-years-has-begun). Wang et al carried out extensive LC‐MS/MS characterization for these vaccine antigens (reference standard, representative material equivalent to cGMP material) to confirm their sequence integrity, their biophysical immunogenicity, glycosylation patterns (both N‐linked and O‐linked), and disulfide linkages. Data from this report demonstrated the immunogenicity of the gp120 antigens, provided comprehensive characterization of the molecules, set the benchmark for assessment of current and future CTM lots, and established the physicochemical groundwork for interpretation of future clinical trial data.^63^

As an example of the applicability of MS‐based methods, trimeric Env glycoproteins commercially manufactured under current good manufacturing practice (cGMP) were analyzed using a hybrid linear trap/Fourier transform ion cyclotron resonance (FTICR) mass spectrometer coupled with a Waters nanoAcquity UPLC system. The BG505.SOSIP.664 HIV‐1 vaccine candidate cGMP was produced from a 200L bioreactor and extensive characterized for molecular structure, disulfide bond patterns, and N‐linked glycosylation profile. This is the first report of a trimeric HIV vaccine clinical trial material produced under cGMP conditions being comprehensively characterized using mass spectrometry. The methods reported in this study should pave the way for the cGMP production of other native‐like Env glycoprotein trimers of various designs.^15^

MS has been instrumental for deciphering the structure of these complex glycoproteins being developed and tested as HIV vaccines as exemplified above. The MS‐ based structural characterization methods provided complementary information to the routinely used analytical methods for in‐process and batch release testing for refining the Env structure during vaccine design.

### 2.2 Influenza

Influenza virus is a segmented, enveloped RNA virus and is among the most virulent pathogens. Within the influenza virus family, there are three genera: A, B and C. Influenza A viruses are further subtyped according to their surface antigens, haemagglutinin (HA) and neuraminidase (NA), of which 18 HA subtypes and 11 NA subtypes have been identified to date and many different combinations of HA & NA are possible (https://www.cdc.gov/flu/avianflu/influenza-a-virus-subtypes.htm). Vaccination against influenza virus is the most effective means of prevention and since 1945 egg‐derived and cell‐derived influenza vaccines have been used as a preventive tool during flu season.

In 1995, the World Health Organization (WHO) recommended developing cell‐derived influenza vaccines and, since then, a number of mammalian cell lines have been evaluated for the manufacture of influenza vaccines.^64^, ^65^, ^66^ In 2012, the FDA approved the first cell culture‐derived influenza vaccine, Flucelvax (Novartis vaccine) and in 2013, the FDA approved Flublok (Protein Sciences Corporation); the first licensed recombinant vaccine using an insect virus expression system (https://www.cdc.gov/flu/protect/vaccine/vaccines.htm). During seasonal epidemics, 5‐15% of the worldwide population is typically infected, resulting in 3‐5 million cases of severe illness and up to 500 000 deaths per year (http://www.who.int/mediacentre/factsheets/2003/fs211/en/).

The unpredictability associated with the strain of influenza virus in circulation is due to antigenic drift (frequent mutations of viral proteins) and, to address this, strain selection is made annually to ensure protection.^67^ Single‐radial immunodiffusion (SRID) is the approved method to measure the content of each hemagglutinin (HA) in the inactivated influenza vaccines. The SRID method to quantify the HA content of influenza vaccines relies on strain‐specific reference reagents the availability of which may cause delays in the formulation of the influenza vaccine especially during a pandemic outbreak. In a WHO workshop organized in 2010 in Ottawa, Canada, it was decided to explore alternative approaches for quantification of HA content to speed up the vaccine delivery process in future responses to pandemic influenza and in seasonal vaccine release.^68^ Various promising methods as alternatives to SRID assay are currently being developed including HPLC and MS. The primary objective is to have a method that can be used to perform quantitation without reference standards, and thus, to formulate virus strains without delay owing to reference reagent availability.^69^

**Figure 5:**
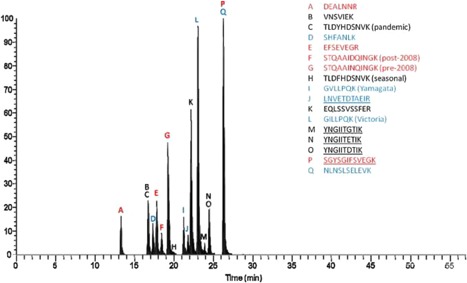
Liquid chromatography‐multiple reaction monitoring (LC‐MRM) chromatogram of a commercial vaccine in which 17 peptide pairs were simultaneously monitored through use of an isotope dilution approach and all MRM transitions were co‐added. The peptides in red are for quantification of H3N2 subtypes, the peptides in black are for quantification of H1N1 subtypes, and the peptides in blue are for the quantification of HA and NA from influenza B. The peptides that are underlined are for NA quantification while those that are not underlined are used for HA quantification. Reprinted from Williams et al, Vaccine, 2012, 30(14), pp. 2475‐2482, Copyright (2018), with permission from Elsevier

MS‐based proteomics methods for measuring a HA strain content are being developed at the Centers for Vaccine Evaluation (CVE) in Canada and the National Center For Environmental Health (NCEH) in the Centers for Disease Control And Prevention (CDC) in the United States. These MS‐based methods are being developed to be used in a quality control lab to simultaneously identify the three (or four) different virus strains and to measure each of the strains in the influenza vaccine. The researchers at NCEH‐CDC have been specifically working on the evaluation of isotope dilution mass spectrometry (IDMS) methods for accurate quantitation of HA in primary influenza standards since 2008. LC‐MS/MS methods involving isotope dilution mass spectrometry (IDMS) in conjunction with multiple reaction monitoring (MRM) have been applied to detect targeted peptides using isotopically labeled peptide standards for the absolute quantification of HA subtypes in the influenza vaccine (Figure 5). The LC‐IDMS‐MRM assay is applicable to trivalent matrices, and can be used without sample cleanup and in the presence of detergents and other proteins. More importantly, the IDMS method was performed without using any strain‐specific virus reference standards for the quantification of HA in the avian influenza H5N1 vaccine. However, direct comparison of results obtained by LC‐IDMS‐MRM and those obtained by SRID was not performed.^70^, ^71^, ^72^

In the spring of 2013, the Chinese Health and Family Planning Commission notified the WHO of the first confirmed human infection with avian influenza (H7N9) virus and LC‐IDMS‐MRM which had been developed to quantify HA and NA in H7N2, H7N7, and H7N9 influenza [A/Netherlands/219/2003 (H7N7) and A/Shanghai/2/2013 (H7N9] was used to analyze these samples to confirm the H7N9 strain.^73^ In a separate study, the HA antigen yields of the vaccine strain [A/Puerto/Rico/8/34 (PR8)] was also determined using LC‐IDMS‐MRM.^74^ The researchers at NCEH‐CDC also demonstrated a unique quantification of immunoreactive viral influenza proteins by immunoaffinity capture followed by IDMS‐MRM (IC‐IDMS‐MRM). The IC‐IDMS‐MRM method is truly an alternative to the approved SRID method as it has dual purpose “potency and content determination”, was found to be equivalent to the SRID method and can also be used in response to a pandemic influenza threat.^75^, ^76^ Additionally, a direct Ultra‐Performance Liquid Chromatography (UPLC)‐IDMS method was reported for the rapid and accurate quantification of influenza NA.^77^ Label‐free MS‐based methods have also been reported for the simultaneous identification and quantification of HA and NA in influenza vaccine with samples analyzed by LC‐MS^E^ on a Waters Synapt G2 mass spectrometer.^78^ Quantification of proteins by label‐free LC‐MS^E^ is a powerful tool, in this method, alternating scans of low collision energy and elevated collision energy during LC‐MS analysis to obtain both protein identity and quantity in a single experiment. Quantification based on the experimental data showed that the signal intensity was proportional to concentration which allowed for the amount of any protein in the mixture to be estimated. LC‐MS^E^ utilizes parallel, multiplex fragmentation where all peptide precursors are simultaneously fragmented throughout the chromatographic separation process regardless of intensity. This allows data‐independent identification of lower abundance peptides and provides increased proteome coverage and dynamic range of protein quantification compared with data‐dependent LC‐MS/MS.^79^, ^80^

The researchers at the Center for Vaccine Valuation (CVE) in Canada were concurrently working to develop chromatography‐based methods for measuring the HA content in the influenza vaccine and developed a number of HPLC methods. Garcia‐Canas et al. reported an on‐line method that combined SEC with RP‐ HPLC to effectively separate 3 subtypes of HA antigens in the trivalent vaccines based on their different elution profiles. However, these methods need antigen reference standards to quantitate the HA antigen.^81^, ^82^ CVE researchers also reported a method for the simultaneous and absolute quantification of HA and NA levels in commercial influenza vaccines and the separated antigens were identified by enzymatic digestion followed by LC‐MS^E^.^83^, ^84^ More recently, a fast and highly selective method to determine HA content in quadrivalent influenza vaccine by reversed‐phase HPLC was reported. This HPLC method showed excellent resolution of all four hemagglutinins and is also the only physicochemical method capable of distinguishing the B strains in quadrivalent influenza vaccines.^85^ The eluents used for this HPLC method were 0.04% (v/v) aqueous trifluoroacetic acid (TFA) and 0.03% (v/v) TFA in 25% ACN and 75% 2‐propanol. With these conditions, the developed method could easily be implemented as an online LC‐MS/MS method to speed data collection and gain additional structural information of the separated HA antigens.

The SRID assay has been used for over 40 years as a quantitative and potency method throughout the world despite issues regarding variability and availability of standard reagents. Thus, even though new methods like HPLC and mass spectrometry are being developed, it will take some time for these methods to be adopted worldwide. As the next influenza pandemic cannot be predicted, the health authorities’ pandemic preparedness efforts include efforts to ensure expedited availability of pandemic vaccines. Methods such as HPLC and MS, with their ability to quantitate antigens without standard reference reagents, can become a cornerstone of pandemic influenza preparedness.

### 2.3 Dengue

The viral genus *Flavivirus*, includes Dengue virus, yellow fever virus, and Zika virus. Dengue represents the most common mosquito‐borne disease in humans and causes ∼400 million cases of infection, ∼500 000 hospitalizations, and ∼12 500 deaths estimated to occur each year.^86^

**Figure 6:**
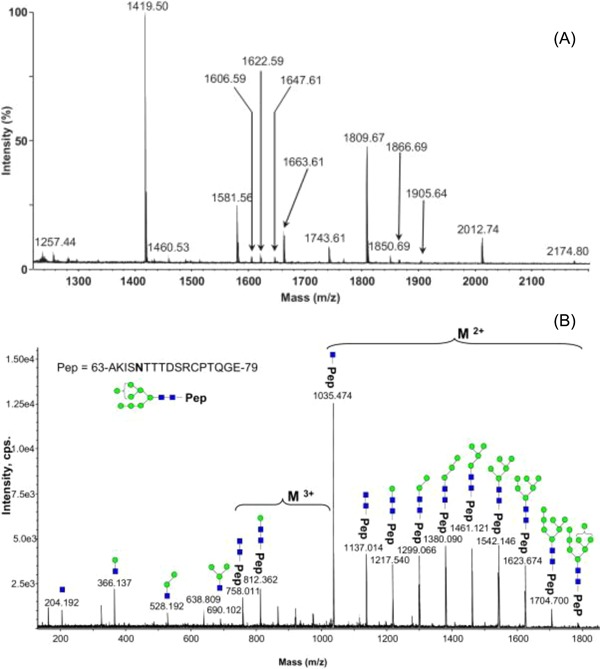
Analysis of the N‐glycan profile of the E‐protein by matrix‐assisted laser desorption/ionization time‐of‐flight mass spectrometries (MALDI‐TOF‐MS). Symbols (blue rectangle, GlcNAc; green circle, mannose). A, N‐glycans present on Sanofi Pasteur tetravalent dengue vaccine (CYD) E‐protein. B, An example of a nanoLC–ESI‐MS/MS spectra of the of the parent ion at *m/z* 1190.15 from CYD E‐protein glycopeptide, provides direct evidence for the high mannose‐type glycosylation linked to Asn67 site. Reprinted from Dubayle et.al, Vaccine, 2015, 33(11), pp.1360‐1368, Copyright (2018), with permission from Elsevier

The world's first dengue vaccine Dengvaxia (CYD‐TDV) developed by Sanofi Pasteur is a live attenuated tetravalent chimeric vaccine made using recombinant DNA technology. The tetravalent dengue vaccine CYD‐TDV produced in mammalian Vero cells consists of four chimeric viruses. The N‐glycans from the Env protein of Dengvaxia were released by in‐gel PNGase F deglycosylation, purified and measured by MALDI‐TOF MS in positive ionization mode using 2,5‐dihydroxybenzoic acid as a matrix. Detected ions were singly charged sodium adducts and monoisotopic masses. The N‐linked glycans of the Env proteins were found to be a mix of high‐mannose, hybrid and complex glycans (Figure 6). Site‐specific N‐glycosylation analysis of Dengvaxia using nanoLC–ESI‐MS/MS demonstrated that both asparagine residues 67 and 153 were glycosylated and, predominately, the N‐glycan at Asn67 was a high mannose‐type and at Asn153 was mainly a combination of complex‐ and hybrid‐type glycans.^87^ This study provided important new insights for the role of glycans in the dengue virus‐host cell interactions.

In a separate study, accurate quantitation of the expressed four viral particles in the tetravalent dengue vaccine (CYD) was performed using targeted MS in selected reaction monitoring (SRM) mode.^88^ The study described an orthogonal quantitation strategy (targeted MS in SRM mode) and demonstrated that the variability of the MS method was low (between 8% and 17%) and the assay was linear between 6.25 and 200 nmol/L. Based on the reported method performance, it could be used to release future batches of the tetravalent dengue vaccine.

### 2.4 Ebola

Ebola hemorrhagic fever is one of the most fatal viral diseases worldwide with the 2014‐2016 Ebola outbreak causing over 28 000 cases of infection and 11 000 deaths. After 40 years, the quest to have an effective vaccine for Ebola virus infections continues. Presently, a number of vaccine candidates are being explored and multiple experimental clinical trials are ongoing.

As evidence of the utility of MS as an important tool in vaccine development, it has recently been used to characterize some of these emerging Ebola vaccine candidates.^89^ In one of the studies, glycan identification in the transmembrane glycoprotein (GP1) and soluble glycoprotein (sGP) of Ebola virus expressed in human embryonic kidney cells (EBOV Yambuku) was performed with normal‐phase HPLC, MALDI‐TOF MS, and negative ion nanoLC‐ESI‐MS/MS. The results showed that most glycans were complex, and sialylation was primarily absent.^90^ More recently, comprehensive characterization of glycosylation was carried out on the N‐linked and O‐linked glycosylation patterns for GP_1,2_ of five pathogenic Ebola viruses (BDBV, SUDV, TAFV, and two EBOV variants) produced in mammalian 293 T cells. The data presented demonstrated that the N‐glycan patterns were similar between GP_1,2_ but O‐glycan patterns were remarkably different on GP_1,2_ the five Ebola viruses. This study should serve as the foundation for future Ebola viral entry and immunogenicity studies.^91^

To improve on the conventional approaches for absolute quantitation of GP1 in Ebola virus‐like particles (eVLPs), an isotope dilution full‐scan liquid chromatography‐high‐resolution mass spectrometry method was developed using an UltiMate 3000 HPLC and an Orbitrap Elite Hybrid Ion Trap‐Orbitrap mass spectrometer.^92^ The reported MS quantitation method provided not only a means to rapidly determine eVLP batch quality based upon quantitation of antigenic GP1 but also ensured adequate preclinical/clinical dosing.

### 2.5 Chikungunya

Chikungunya is a mosquito‐borne viral disease, endemic in Africa and Southeast Asia and has also recently emerged in the Caribbean. Currently there are no drugs or vaccines available for treatment or prevention. The name “Chikungunya” derives from a Makonde word meaning “to become contorted,” and describes the stooped appearance of sufferers with joint pain (arthralgia). There are continuing efforts to develop a vaccine for Chikungunya, and currently a clinical trial of experimental vaccines to prevent infection with Chikungunya virus is ongoing.^93^

MS‐based methods have been used to characterize the experimental Chikungunya (CHIKV) virus‐like particle (VLP) vaccine. In one of the studies, a RP‐HPLC method was developed to separate capsid, E1, and E2 proteins in CHIKV VLP vaccine with good resolution with the separated protein components verified by MALDI‐TOF MS. In the same study, the post‐translational modifications on the viral glycoproteins E1 and E2 were further identified by intact protein mass measurements with LC‐MS. The RP‐LC‐MS method was used, in addition to characterizing the PTMs, for monitoring both the product purity during process development and assessing product stability during formulation development.^94^ In another study a HILIC LC method with fluorescence MS detection was performed to characterize the *N*‐glycosylation patterns of CHIKV VLPs. The developed method was used to monitor glycosylation during CHIKV VLPs vaccine process development to ensure batch consistency.^95^

### 2.6 Meningococcal

*Neisseria meningitides* (meningococcus) is a gram‐negative bacterium and causes meningitis and other forms of meningococcal disease such as meningococcemia; a life‐threatening sepsis. There are two types of FDA‐approved vaccines for protection against meningococcal disease; meningococcal conjugate vaccines (*Menveo®* and *Menactra®* are quadrivalent vaccines for serogroups A, C, W135, and Y) and serogroup B meningococcal vaccines (*Bexsero®* and *Trumenba®*). MS‐based approaches have been used to perform the physicochemical characterization for all of these vaccines.^96^ In one of the studies, researchers used LC‐MS to characterize glycosylated lysine residues in *Menveo*®.^97^ In another study a LC‐MS method was used to determine the relative reactivity of lysine residues in CRM_197_ to determine which of these amino acids were more susceptible to conjugation.^98^ LC‐MS was also used to quantify the *Bexsero®* vaccine which was the first vaccine developed by reverse vaccinology; a genome‐based approach to vaccine development.^99^ Tani et al. used a Hi3 label‐free LC‐MS^E^ methodology to perform quantification of outer membrane vesicle proteins of the *Bexsero*® vaccine. The Hi3 approach uses the intensity of the MS signal response for the three most intense tryptic peptides to perform absolute quantitation.^80^ The three recombinant proteins in the *Bexsero*® vaccine are the active components and Hi3 label‐free LC‐MS^E^ methodology was used to qualitatively and quantitatively characterize each of these three proteins. The Hi3 MS method was performed as an additional characterization in addition to the standard quality control testing for vaccine batch release.^100^

*Trumenba®* is also a well‐characterized vaccine, composed of two factor H binding protein (fHbp) variants that were recombinantly expressed in *E. coli* as native lipoproteins: rLP2086‐A05 and rLP2086‐B01. Gas chromatography‐mass spectrometry (GC/MS) and liquid chromatography‐mass spectrometry (LC/MS) were used to analyze the composition of fatty acids released from rLP2086‐A05 and rLP2086‐B01 and for complete characterization of the primary structure of both recombinant lipoproteins. The LC‐MS analysis was carried out using an Acquity UHPLC/UV system interfaced to an ultrahigh‐resolution electrospray ionization quadrupole time‐of‐flight mass spectrometer (Bruker Daltonics maXis).^101^

### 2.7 Role of mass spectrometry in the other vaccine candidates

Following are some of the other literature reports that also illustrated the role of MS‐based techniques during vaccine development and manufacturing.

#### 2.7.1 Hepatitis E virus (HEV)

*Hecolin*®, *Innovax* was the first VLP‐based vaccine against hepatitis E virus (HEV) obtained from *E. coli* and was licensed by the FDA of China in 2011. This VLP vaccine consists of a truncated, recombinant HEV capsid protein (p239) expressed in *E. coli* that spontaneously forms a VLP particle of ∼20‐30 nm in diameter.^102^ Several analytical methods including LC‐MS peptide mapping were employed to examine the quality, size and stability of the p239 protein comprising this VLP vaccine. To ensure consistency in terms of size and quality, multiple batches of commercial scale p239 containing VLPs were characterized by Size Exclusion Chromatography and LC‐MS methods.^103^

#### 2.7.2 Human papillomavirus (HPV)

*Cervarix*®, is a Human papillomavirus (HPV) types 16 and 18 vaccine licensed in 2009. HPV infection can cause the development of cervical cancer, which is the second most common cause of death in women worldwide from cancer.^104^ Although multiple types of HPVs can lead to cervical cancer in humans, types 16 and 18 are responsible for ∼70% of cases.^105^ LC‐MS peptide mapping was performed to confirm the primary sequences of the recombinant L1 protein which is the major antigenic protein of the capsid of HPV types 16 and 18. A peptide sequence coverage of ∼97% was observed for both HPV 16 and 18 VLPs. Additionally, one post‐translational modification, N‐terminal acetylation, was also identified in the LI viral proteins within the VLPs. The LC‐MS peptide mapping was used to verify the batch to batch consistency and identify post‐translational modifications that can occur during protein expression and purification.^104^, ^106^

#### 2.7.3 Nipah virus (NiV)

No therapeutics or vaccines are currently approved for use in humans against *Nipah virus* (NiV), an emerging, highly pathogenic, zoonotic virus from the *Paramyxoviridiae* family. Soluble glycoprotein and virus vector platforms are being developed for protection against NiV but the sporadic nature of NiV outbreaks makes large scale Phase III clinical trials difficult to plan.^107^ In a very interesting recent application of MS in the field of vaccines, Vera‐Velasco et al not only quantified the viral F, G and M proteins present in the viral particles but also analyzed the cellular proteomic composition in the NiV vaccine candidate. Traditionally, viral particles have been described as pure entities carrying only viral‐derived proteins but the authors successfully analyzed the ratio between cellular and viral proteins in the NiV vaccine candidate using LC‐MS/MS.^108^

#### 2.7.4 West Nile vaccine (WNV)

West Nile fever is a flavivirus that causes a viral infection typically spread by mosquitoes. To date, no vaccine is available to prevent the West Nile infection. Partially purified virus‐like particles were resolved by SDS‐PAGE, and the Coomassie blue‐stained band corresponding to Env protein was excised from the gel, destained, and analyzed by MS. Microcapillary LC‐MS was performed using a LCQ DECA ion‐trap mass spectrometer (Thermo Finnigan) to identify the presence of Env protein in virus‐like particles to help in developing a potential vaccine.^109^

#### 2.7.5 Enterovirus vaccine (EVs)

In December 2015, China's Food and Drug Administration approved two inactivated EV‐A71 vaccines for preventing severe HFMD (hand, foot, and mouth disease). LC‐MS/MS peptide mapping and N‐ and C‐terminal sequencing was performed using a Q‐Exactive mass spectrometer. The molecular weights of VP0, VP1 and VP3 capsid proteins of EV71 VLPs, were determined using a MALDI‐TOF/TOF MS 4800 Proteomics Analyzer (Applied Biosystems, Framingham, MA).^110^

## 3 REGULATORY CHALLENGES IMPLEMENTING MASS SPECTROMETRY IN THE QUALITY CONTROL LABORATORY

MS is a technique of choice for the identification and structural characterization of tremendously complex glycoproteins and is a routinely used tool during the early research and development of protein‐based molecules. Implementation of MS methods in chemistry, manufacturing and controls (CMC) and quality control environments for vaccines has been of longstanding interest but has faced regulatory challenges. To the best of our knowledge, the authors are not aware of any LC‐MS‐based release method used for any vaccine product in the quality control/cGMP environment.

There is always some reluctance to implement new methods in the quality control/cGMP environment and, in the case of MS, there are multiple misconceptions regarding the technique. Although MS is not without limitations, some of the misconceptions relating to reproducibility and validation are overstated. To implement a method in the QC environment, the method has to be validated to demonstrate the method's accuracy, precision, intermediate precision, specificity, linearity, limit of detection, and limit of quantitation. Furthermore, appropriate system suitability controls or calibrators are part of the method to ensure repeatability. All of these parameters and controls ensure that during the performance of the assay, no variability between different instruments and operators arises. Unfortunately, some aspect of these validation activities are inherently challenging for a MS‐based method. Thus, the burden of proof lies with the vaccine developer to convince a regulator that the MS‐based method is a phase appropriate release test without regulatory guidelines applicable to this class of drugs. It is clear from the author's perspective that classical validation regulations as stated in the ICH guidelines are unsuitable for MS‐based methods. In the last decade, some of the conventional gel‐based or plate‐based release assays have been replaced by HPLC or capillary electrophoresis (CE) methods in the QC environment. These HPLC‐ or CE‐based assays are used to monitor aggregation, fragmentation, clipping, and for content determination. As the application of MS in the vaccine QC environment is an evolving story, the challenge of convincing regulators should be directed toward highlighting MS capabilities as has been shown in the multiple examples in this manuscript. For those working in this field, it will be important to present MS data testing multiple quality attributes of the sample in the same analytical run in combination with HPLC/CE methods or by performing MS analysis to validate simpler HP/UPLC assays used to assess batch to batch consistency. In this way, comparative data sets could demonstrate what the authors believe is the ultimate utility of MS‐based methods for QC release of vaccines. The main rational for the MS‐based methods in the QC/cGMP environment should not be to replace the existing methods. Rather, it should be a supplemental batch release test to ensure that the product is within specifications and without any unwarranted modifications. Using MS techniques will ensure improved quality and increased safety for clinical trial participants.

## 4 CONCLUSION

In conclusion, the evidence suggests that the emergence of MS‐based techniques has advanced the field of vaccine development. LC coupled with MS represents a powerful characterization tool in vaccine development; especially for the recombinantly produced antigens. LC‐MS techniques are rapidly being used not only to quantitate vaccine antigens and explore molecular structure but also for the comprehensive analysis of glycans. An important question remains on how to establish the MS techniques in the quality control cGMP labs for vaccine release given the wide use of classical methods and lack of regulatory guidance. Nevertheless, after a decade of steady partnership with vaccine developers, MS has expanded from a niche application to an enabling platform for vaccine development.
